# Interactions of FK506 and Rapamycin With FK506 Binding Protein 12 in Opportunistic Human Fungal Pathogens

**DOI:** 10.3389/fmolb.2020.588913

**Published:** 2020-10-16

**Authors:** Sandeep Vellanki, Alexis E. Garcia, Soo Chan Lee

**Affiliations:** South Texas Center for Emerging Infectious Diseases, Department of Biology, The University of Texas at San Antonio, San Antonio, TX, United States

**Keywords:** FK506, FKBP12, rapamycin, calcineurin, TOR, fungi, antifungal

## Abstract

Over the past few decades advances in modern medicine have resulted in a global increase in the prevalence of fungal infections. Particularly people undergoing organ transplants or cancer treatments with a compromised immune system are at an elevated risk for lethal fungal infections such as invasive candidiasis, aspergillosis, cryptococcosis, etc. The emergence of drug resistance in fungal pathogens poses a serious threat to mankind and it is critical to identify new targets for the development of antifungals. Calcineurin and TOR proteins are conserved across eukaryotes including pathogenic fungi. Two small molecules FK506 and rapamycin bind to FKBP12 immunophilin and the resulting complexes (FK506-FKBP12 and rapamycin-FKBP12) target calcineurin and TOR, respectively in both humans and fungi. However, due to their immunosuppressive nature these drugs in the current form cannot be used as an antifungal. To overcome this, it is important to identify key differences between human and fungal FKBP12, calcineurin, and TOR proteins which will facilitate the development of new small molecules with higher affinity toward fungal components. The current review highlights FK506/rapamycin-FKBP12 interactions with calcineurin/TOR kinase in human and fungi, and development of non-immunosuppressive analogs of FK506, rapamycin, and novel small molecules in inhibition of fungal calcineurin and TOR kinase.

## Introduction

Opportunistic invasive fungal pathogens cause significant morbidity and mortality in immunocompromised patients such as those undergoing stem cell or organ transplantation and in individuals with a dysfunctional immune system due to diabetes mellitus or AIDS (Husain et al., 2003; Enoch et al., 2006; Person et al., 2010; Low and Rotstein, 2011; Wang et al., 2017). While the most common opportunistic agents of fungal infections are *Aspergillus fumigatus*, *Cryptococcus neoformans*, and *Candida albicans*, there has been a rising number of cases caused by other fungi such as those belonging to Mucorales (Pfaller and Diekema, 2004). Unlike human cells, these fungi have a cell wall to maintain homeostasis which makes it an attractive target for antifungals such as echinocandins (Cortés et al., 2019; Lima et al., 2019). Other antifungals such as azoles or polyenes target ergosterol in the cell membrane (Maertens, 2004). There is increasing evidence that fungi are either intrinsically resistant to antifungals or can develop resistance after prolonged exposure to antifungals (Wiederhold, 2017). There has also been less than optimistic progress in the development of antifungal drugs in the past decade. Therefore, there is an urgent need for the development of new antifungals to combat deadly fungal infections.

**FIGURE 1:**
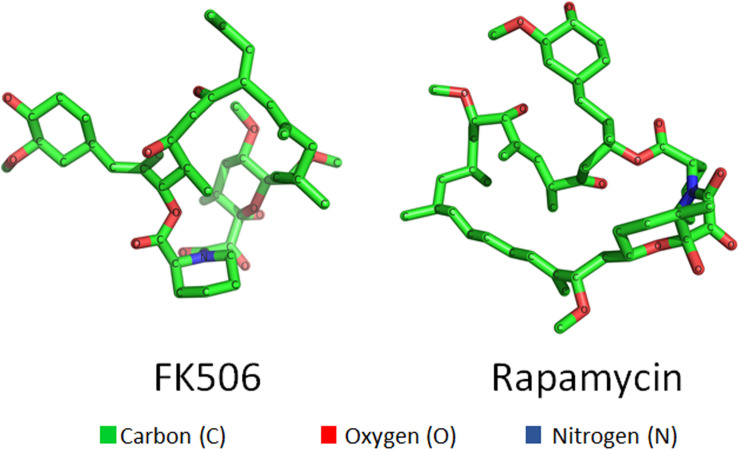
Three dimensional structures of small molecules—FK506 **(left)** and rapamycin **(right)**.

**FIGURE 2:**
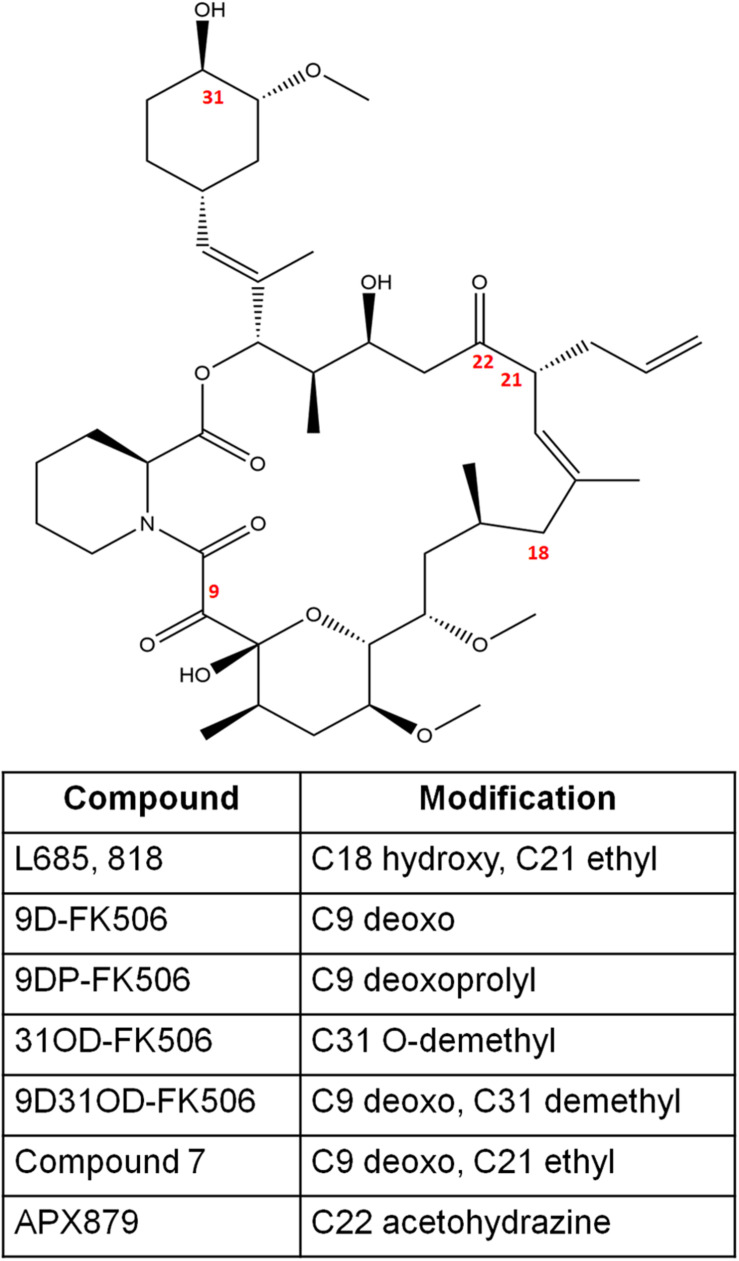
The chemical structures of FK506 and its analogs discussed in this study. The numbers in red font indicate the modified positions to generate analogs.

Small molecules are compounds targeting a specific biological component or a function. These small molecules which are either new compounds or approved drugs have been investigated for antifungal activity (Cully, 2018; Garcia et al., 2018; Bechman et al., 2019; Zarakas et al., 2019). One such small molecule is FK506 (tacrolimus; Figures 1, 2), a 23-membered macrolide lactone first identified from the fermentation broth of the bacteria *Streptomyces tsukubaenis* by Fujisawa Pharmaceuticals (Kino et al., 1987). FK506 is an immunosuppressive drug used for the prevention of graft rejection in patients undergoing organ transplantation (Thomson, 1989; Meier-Kriesche et al., 2006; Ban et al., 2016). The immunosuppression by FK506 results from inhibition of T-cell activation and proliferation (Schreiber and Crabtree, 1992). On entering the cell, FK506 binds to a prolyl isomerase FKBP12 in the cytosol and the FK506-FKBP12 drug-immunophilin complex binds and inhibits the activity of calcineurin, a calcium-calmodulin dependent protein phosphatase. Calcineurin governs the activation and translocation of the nuclear factor of activated T cells (NFAT) from the cytoplasm to the nucleus to activate genes such as *Il-2* that are involved in T-cell activation and proliferation (Liu et al., 1991; Schreiber and Crabtree, 1992). When calcineurin activity is inhibited by FK506, the NFAT transcription factor cannot translocate into the nucleus (Schreiber and Crabtree, 1992). Interestingly, calcineurin is not only conserved across other eukaryotes like fungi but is also a key virulent factor in many pathogenic fungi, suggesting that FK506 can be effective against human pathogenic fungi (Steinbach et al., 2007). It was first reported in *C. neoformans* that the mutants lacking calcineurin do not survive in conditions that mimic the host environment (Steinbach et al., 2007). Further studies have shown that calcineurin is also required for virulence and pathogenicity in *C. albicans* (Blankenship et al., 2003; Blankenship and Heitman, 2005), *A. fumigatus* (Steinbach et al., 2006), and *Mucor circinelloides* (Lee et al., 2013; Lee et al., 2015; Vellanki et al., 2020). Although, calcineurin is an attractive target for antifungal drug development, there are two key problems. First, the human and fungal calcineurin share 80% homology, and also fungal and human FKBP12s share about 48–58% sequence homology (Gobeil et al., 2019). The cross-reactivity of FK506 against human and fungal calcineurins represents a major hurdle. To overcome this, it is important to identify regions of calcineurin and FKBP12 that are present in fungi but not in humans. Secondly, FK506 exhibits antifungal activity by inhibiting fungal calcineurin (Odom et al., 1997a; Steinbach et al., 2004), but FK506 is an immunosuppressive drug, therefore it cannot be used in its current form to treat patients with fungal infections. The non-immunosuppressive analogs of FK506 offer a potential solution to overcome this problem. The first part of the review focuses on—(a) structural and molecular interactions between FK506-FKBP12-calcineurin in pathogenic fungi compared to human counterparts which in turn will inform the extent to which fungal FKBP12 and calcineurin are suitable antifungal candidates, (b) non-immunosuppressive analogs of FK506 as potential antifungal drugs.

Rapamycin (Figures 1, 5) is a 29 membered macrolide lactam metabolite produced by the bacteria *Streptomyces hygroscopicus* isolated by Sehgal et al. (1975). Rapamycin was found to inhibit the growth of *S. cerevisiae* and, interestingly, FK506 antagonized the effect of rapamycin, suggesting that FK506 and rapamycin share the same cellular receptor, FKBP12 (Heitman et al., 1991). Indeed, Heitman et al. (1991, 1993) verified that rapamycin forms a complex with FKBP12 and inhibits TOR (target of rapamycin) kinase. Rapamycin was further investigated as a possible antifungal agent with potent activity against *C. albicans* however, it was later discovered that rapamycin possessed potent immunosuppressive activity (Martel et al., 1977; Hwang et al., 2017). This is attributed to rapamycin inhibiting the activation of T-cells and B-cells via the reduction of their sensitivity to interleukin-2 through inhibition of the mammalian Target of Rapamycin (mTOR) (Saunders et al., 2001). Thus, rapamycin has been previously used to prevent organ transplant rejection via immunosuppression (Martel et al., 1977; Saunders et al., 2001; Yoon et al., 2017). The mode of action by which rapamycin inhibits the immune system begins with binding to the prolyl isomerase FKBP12 (Saunders et al., 2001; Yoon et al., 2017). The rapamycin-FKBP12 complex will then bind with mTOR and inhibit its function (Saunders et al., 2001; Yoon et al., 2017). The result is the inhibition of T-cell and B-cell activation leading to immunosuppression. With regards to fungi, the TOR pathway is known to be involved in the response to nutrient resource availability (Cardenas et al., 1999; Bastidas et al., 2012; Nguyen et al., 2020). The serine/threonine kinase TOR is known to interact with two complexes known as TORC1 and TORC2, both of which are known to regulate their targets via phosphorylation (Cardenas et al., 1999; Bastidas et al., 2012). TORC1 is known to control various cellular processes such as protein synthesis, mRNA synthesis and degradation, autophagy, and nutrient transport; while TORC2 is involved in cell wall integrity and actin polarization (Kubota et al., 2003; Bastidas et al., 2012). TORC1 is the only of the two complexes to be sensitive to rapamycin via a similar pathway involving rapamycin binding to FKBP12 forming a rapamycin-FKBP12 complex that binds to TOR resulting in its inhibition (Bastidas et al., 2012; Nguyen et al., 2020). However, a chronic treatment with rapamycin can not only inhibit TORC1 but also disrupts TORC2 *in vivo* (Lamming et al., 2012). In the yeast fungus *Saccharomyces cerevisiae*, inhibition of TOR via rapamycin exposure results in multiple cellular responses such as protein synthesis inhibition and autophagy triggered by nutrient depletion (Cardenas et al., 1999; Bastidas et al., 2012; Nguyen et al., 2020). Therefore, TOR represents a critical target for the development of new antifungals. The second part of the review further highlights rapamycin-FKBP12-TOR interactions in pathogenic fungi.

## Interactions Between FK506, FKBP12, and Calcineurin in Pathogenic Fungal Species

**FIGURE 3:**
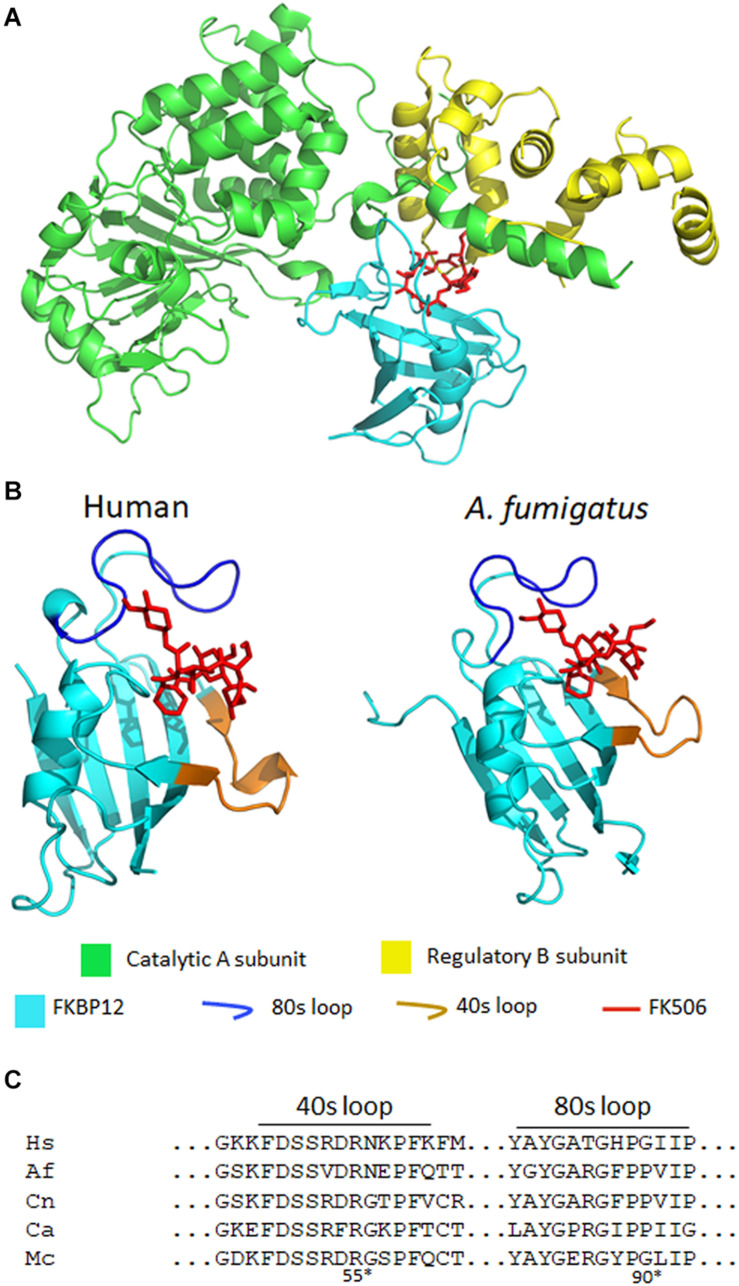
**(A)** A crystal structure of *A. fumigatus* calcineurin A and B, FKBP12, and FK506 [protein data bank (PDB): 6TZ7]. Calcineurin is a protein phosphatase comprising of a catalytic (green) and a regulatory subunit (yellow). The complex of FK506 (red) and FKBP12 (cyan) interacts with the calcineurin heterodimer to form a ternary complex and inhibit calcineurin function. **(B)** In the ribbon diagrams of human (left) and *A. fumigatus* (right) FKBP12-FK506, β sheets wrap around the α helix and the extended loops (such as the 80 and 40s loops) surround the FK506 binding pocket. Left: FK506-HsFKBP12 (PDB: 1FKJ), right: FK506-AfFKBP12 (PDB: 6TZ7). **(C)** Alignment of amino acids of the 40 and 80s loops of FKBP12. Hs, human; Af, *A. fumigatus*; Cn, *C. neoformans*; Ca, *C. albicans*; Mc, *M. circinelloides*. *Residue numbers are in respect to AfFKBP12.

Calcineurin is a heterodimer comprising of a catalytic A subunit and a regulatory B subunit; both subunits are required for its phosphatase activity (Rusnak and Mertz, 2000). The FK506-FKBP12 drug-receptor complex binds at the interface of the calcineurin B regulatory and catalytic A subunit and occludes substrate access (Liu et al., 1991). Thus, the formation of the FK506-FKBP12-calcineurin tertiary complex (Figure 3A) is required for complete inhibition of calcineurin function. Here we detail how calcineurin plays a role in virulence and pathogenesis and how FK506, FKBP12, and calcineurin interact in pathogenic fungi.

### Aspergillus fumigatus

*Aspergillus fumigatus* is the most frequent causative agent of invasive aspergillosis in immunocompromised patients (Brakhage et al., 2010; Baldin et al., 2015). Current treatment for aspergillosis primarily relies on the use of azoles, but polyenes and echinocandins are also used (Maschmeyer et al., 2007; Walsh et al., 2008). With rising antifungal resistance, demand for new antifungal drugs, and alternative treatment are on the rise (Howard et al., 2009; Wu et al., 2020). Previous studies have shown that calcineurin is required for cell wall integrity, hyphae formation, echinocandin resistance, virulence, and others in *A. fumigatus* (Steinbach et al., 2006; Cramer Jr., Perfect et al., 2008; Fortwendel et al., 2010; Juvvadi et al., 2014). Calcineurin inhibitor—FK506 exhibits antifungal activity *in vitro* (Steinbach et al., 2004). In a systemic model of murine aspergillosis, the infected mice treated with FK506 exhibited higher survival rates compared to the infected groups that did not receive FK506 treatment (High and Washburn, 1997). Together the data suggest that calcineurin is an attractive target for aspergillosis.

In *A. fumigatus*, FKBP12 is also the cellular receptor for FK506. Falloon et al. (2015) identified four orthologs of human FKBP12 (HsFKBP12) in *A. fumigatus*: *fkbp12-1, fkbp12-2, fkbp12-3, and fkbp12-*4. It was determined that *A. fumigatus* FKBP12-1 (AfFKBP12) was the binding partner for FK506. The AfFKBP12 shares 55% sequence similarity with HsFKBP12 (Falloon et al., 2015; Gobeil et al., 2019). The core structure of FKBP12 is conserved in both humans and fungi, and it comprises of five to six β sheets (β1–β6) that wrap around the central α helix (Figure 3B; Tonthat et al., 2016). Additionally, three extended loops surround the FK506 binding pocket—40s loop (between β2 and β3), the 50s loop (between β3′ and α1), and the 80s loop (between β4 and β5) (Figure 3B; Tonthat et al., 2016). Interestingly, when AfFKBP12 is present in the apo form, the 80s loop from one monomer docks into the active site of the second monomer and vice-versa to form a dimer possibly through self-catalysis function (Tonthat et al., 2016). Sequence alignment further revealed the presence of a specific proline residue at the tip of the 80s loop facilitates dimerization in AfFKBP12 but not in HsFKBP12 (Figures 3B,C). The self-substrate region overlaps with the FK506 binding region because alteration of the proline residue at the tip of the 80s loop to glycine conferred resistance to FK506 (Tonthat et al., 2016).

To evaluate the role of the 40s and 50s loop in AfFKBP12 interaction with FK506 and calcineurin, Juvvadi et al. (2020) induced amino acid point mutations in 40s (F37M/L) and 50s (W60V) loop. They found that in comparison to the 80s loop point mutations (P90G or V91C), alteration in the 40 and 50s loop increased contacts in the 80s loop resulting in decreased calcineurin binding and higher resistance to FK506 (Juvvadi et al., 2020). Although fungal and human FKBP12 share a striking similarity, there are non-conserved motifs in the 40s and 80s loop of fungal FKBP12 (Figures 3B,C; Juvvadi et al., 2019). Amino acid substitutions in the 40s loop (F22T, Q50M, and R55E) and 80s loop (F88H) impacted the FK506-FKBP12 interaction with calcineurin. Particularly, replacing Phe with His (F88H) resulted in a significant increase in resistance to FK506 due to reduced binding of FK506-FKBP12 complex with calcineurin (Juvvadi et al., 2019). Interestingly, while H88 in the 80s loop is critical for mammalian FK506-FKBP12-calcineurin interaction, F88 is involved in *A. fumigatus* FK506-FKBP12-calcineurin interaction and inhibition of calcineurin function (Figure 3C; Juvvadi et al., 2019). Despite the differences, it is evident that alterations in the 80s loop can adversely affect the binding of both human and fungal FK506-FKBP12 complex to calcineurin (Tonthat et al., 2016; Juvvadi et al., 2019).

### Candida albicans

*Candida albicans* is a commensal organism and can cause deep systemic infections in immunocompromised patients (Stelzner, 1990; Pfaller and Diekema, 2007; Low and Rotstein, 2011; Neville et al., 2015). Current treatments involve three different classes of antifungal drugs including azoles, echinocandins, and polyenes (Pierce et al., 2013; Vila et al., 2017). Antifungal drug resistance in *C. albicans* is not uncommon and is on the rise (Monroy-Pérez et al., 2016; Whaley et al., 2016).

Previous studies have shown that calcineurin is required for survival in serum and consequently, the calcineurin mutants failed to colonize tissues in infected murine animals (Blankenship et al., 2003; Blankenship and Heitman, 2005). Additionally, calcineurin is also required for tolerance to antifungal agents (Sanglard et al., 2003). Therefore, calcineurin represents a suitable target for candidiasis treatment, and understanding the structural differences between FKBP12 and calcineurin in *C. albicans* to humans is critical in developing non-immunosuppressive FK506 analogs.

It was previously reported that similar to *A. fumigatus*, the core structure of *C. albicans* FKBP12 (CaFKBP12) comprises of five β sheets that wrap around the central α helix and also has three extended loops (the 40s, 50s, and 80s) surrounding the FK506 binding pocket (Tonthat et al., 2016). Consistent with the observation made in *A. fumigatus*, the proline residue present at the tip (P104) of 80s loop docks into the active site of adjacent subunit and vice-versa facilitating FKBP12-FKBP12 interaction in apostate (Tonthat et al., 2016). The 80s loop bind in the same pocket as FK506. Interestingly, in *C. albicans* all the captured FKBP12s have P104 in *cis* conformation while in *A. fumigatus* the P90 in one subunit was bound in *cis* conformation while the P90 in other subunit was bound in a *trans*-state (Figure 3C; Tonthat et al., 2016).

### Cryptococcus neoformans

*Cryptococcus neoformans* is the etiologic agent of cryptococcosis (Idnurm et al., 2005). Cryptococcosis primarily affects lungs in immunocompromised patients and can also cause lethal meningitis and encephalitis (Idnurm et al., 2005; Mednick et al., 2005; Bretaudeau et al., 2006). Currently, cryptococcosis can be treated with an azole such as fluconazole, but cryptococcal meningitis may require the use of polyenes such as amphotericin B (Saag et al., 2000). Clinically, the combination of amphotericin B with flucytosine has resulted in better outcomes than treatment with amphotericin B alone (Larsen et al., 1990; Dromer et al., 2008). Regardless, there have been several reports of antifungal resistance in *C. neoformans* (Friese et al., 2001; Lortholary et al., 2006; Cheong and McCormack, 2013; Smith et al., 2015) indicating a need for the development of alternative treatment strategies.

FK506 is toxic to *C. neoformans* at 37°C, but not at 24°C (Odom et al., 1997a,b), thereby suggesting that calcineurin is required for growth at higher temperatures. As a consequence, the calcineurin mutants are not virulent in murine models of cryptococcal meningitis (Odom et al., 1997b). Cruz et al. identified the homolog of FK506 binding partner FKBP12 in *C. neoformans*. They found that CnFKBP12 share 59 and 57% sequence similarity with the HsFKBP12 and CaFKBP12, respectively. The amino acid residues that form the FK506-binding pocket were highly conserved across species. A single amino acid substitution in this conserved site—W60R destabilized CnFKBP12 and rendered resistance to FK506 and rapamycin (Cruz et al., 1999). Consistent with the findings in *C. albicans* and *A. fumigatus*, CnFKBP12 also has conserved phenylalanine in the 80s loop (F88) which is absent in HsFKBP12 (Figure 3C; Juvvadi et al., 2019).

### Mucor circinelloides

Mucormycosis is an emerging lethal infection caused by fungi of order Mucorales (Petrikkos et al., 2012). *M. circinelloides* is one of the causative agents of this deadly disease (López-Fernández et al., 2018; Vellanki et al., 2018). When *M. circinelloides* is grown in the presence of FK506 it exhibits yeast growth instead of hyphae which shows that calcineurin regulates dimorphism in this fungus (hyphal to yeast transition) (Lee et al., 2013). Mutants lacking functional calcineurin are less virulent than the wild type which suggests that calcineurin is a key target for the treatment of mucormycosis (Lee et al., 2013, 2015; Vellanki et al., 2020). *M. circinelloides* encodes three calcineurin catalytic subunits (CnaA, CnaB, and CnaC) and one regulatory subunit (CnbR) (Lee et al., 2013). We have previously reported that mutations in the FK506-FKBP12 binding region of CnaA and the latch region of CnbR confer resistance to FK506 (Lee et al., 2013, 2015). Specifically, the CnbR mutation N125Y disrupted CnbR-FKBP12 interaction due to steric clashes with residue F47 and Q48 of McFKBP12 (Lee et al., 2015). The CnaA residue W377 is required for FK506-CnaA hydrophobic interaction and spontaneous mutation resulting in W377L was predicted to disrupt the hydrophobic pocket at the binding interface (Lee et al., 2015).

Gobeil et al. (2020) found that the amino acid sequence of McFKBP12 is 58% identical to HsFKBP12. The sequence variations were present in the 40, 50, and 80s extended loops surrounding the FK506 binding pocket. Interestingly, McFKBP12 sequence is 65% identical to AfFKBP12 and share structural similarity but functionally they are not equivalent in inhibiting calcineurin function (Gobeil et al., 2020). When *Affkbp12* was replaced with *Mcfkbp12* in *A. fumigatus*, FK506 was not able to inhibit calcineurin function. As mentioned above, in the presence of FK506 the F88 residue in the 80s loop of AfFKBP12 is critical in the FK506-AfFKBP12-calcineurin interaction. Interestingly, McFKBP12 possesses a tyrosine at the 88^th^ residue instead of phenylalanine because of which McFKBP12-FK506 failed to inhibit calcineurin function in *A. fumigatus* (Figure 3C; Gobeil et al., 2020).

### Efficacy of FK506 Analogs Against Pathogenic Fungi

Fungal infections are prevalent in immunocompromised patients, therefore FK506 cannot be used as an antifungal to treat patients. To circumvent this problem several attempts have been made in generating FK506 analogs that have a lesser binding affinity toward human calcineurin (to reduce immunosuppression) while retaining antifungal properties.

L685,818 (Figure 2) is an FK506 analog and antagonist (Dumont et al., 1992). It is a C18 hydroxy, C21ethyl derivative of FK506. The presence of the C18 hydroxy group prevents L685,818-HsFKBP12 from inhibiting bovine calcineurin, and subsequently, L685,818 was found to be significantly less immunosuppressive (Dumont et al., 1992; Rotonda et al., 1993). Unlike, HsFKBP12 the yeast FKBP12 bound to L685,818 can inhibit bovine calcineurin function (Rotonda et al., 1993), and therefore its antifungal efficacy was evaluated. The analog L685,818 inhibited the growth of *C. neoformans* at 37°C, however, its potency was 10–100 times lower than FK506 (Odom et al., 1997a). Also, L685,818 alone or in combination with caspofungin did not exhibit antifungal activity against *A. fumigatus* (Kontoyiannis et al., 2003).

Nambu et al. (2017), synthesized FK506 antagonists which are permeable in mammalian cells but not in fungal cells, this way a dichotomy of FK506 and the antagonist will result in an inhibition of fungal calcineurin only. The antagonist compounds 15 and 18 (modifications shown in Figure 2) in combination with FK506 significantly reduced *A. fumigatus* proliferation compared to a challenge with antagonist alone.

FK506 analogs: 9-deoxo-FK506 (9D-FK506), 9-deoxo-prolyl-FK506 (9DP-FK506), 31-O-demethyl-FK506 (31OD-FK506), and 9-deoxo-31-O-demethyl-FK506 (9D31OD-FK506) (Figure 2) were generated by manipulating FK506 biosynthetic genes in *Streptomyces* spp. (Ban et al., 2013; Shinde et al., 2015). These analogs showed a significantly less immunosuppressive effect *in vitro* than FK506 (Lee et al., 2018). All the analogs except 9DP-FK506 exhibited antifungal activity against *C. neoformans*, *A. fumigatus*, and *C. albicans in vitro*, however, their efficacy was lower when compared to FK506. In a murine model of systemic cryptococcal infection, the infected mice treated with 9D31OD-FK506 and fluconazole exhibited prolonged survival when compared to the infected mice treated with either of the agents alone. This confirms that an *in vivo* synergistic interaction occurs between FK506 analog and fluconazole (Lee et al., 2018).

In another study, Beom et al. (2019) generated seven new FK506 analogs with modifications in either FKBP12 or calcineurin binding sites. The 9-deoxo-21-ethyl-FK506 analog (named as compound 7 in the reference; Figure 2) exhibited significantly higher antifungal activity against *C. albicans* and *A. fumigatus* when compared to the previously described analog 9D31OD-FK506. This new analog also exhibited synergistic antifungal activity with fluconazole against *C. neoformans* (Beom et al., 2019).

A comparison of fungal and human FK506-FKBP12-calcineurin ternary structures revealed key differences between the species (Juvvadi et al., 2019). While a His 88 at the interface of FK506-HsFKBP12 is required for productive binding and inhibition of human calcineurin, Phe 88 on fungal FKBP12 is required to inhibit fungal calcineurin (Figure 3C). A Val 91 residue at the interface of fungal FK506-FKBP12 is also not conserved in mammalian counterparts. These differences were exploited to generate an FK506 analog—APX879, characterized by an acetohydrazide substitution at C22-ketone. The APX879 (Figure 2) interacts less favorably than FK506 with HsFKBP12 due to a higher steric clash with H88 residue (Juvvadi et al., 2019; Gobeil et al., 2020). Subsequently, APX879 was found to be 71-fold less immunosuppressive than FK506, as measured by their ability to induce IL-2 production from differentiated CD4^+^ T cells. APX879 exhibits antifungal activity against *C. neoformans*, *C. albicans*, *A. fumigatus*, and *M. circinelloides*, however, its efficacy is lower than FK506 *in vitro*. Interestingly, APX879 is less toxic than FK506 in a murine model of cryptococcal infection. Additionally, infected animals treated with a combination of APX879 and fluconazole exhibited significantly extended median survival rates compared to infected animals treated with APX879 or fluconazole alone. The APX879 was not efficacious in murine models of aspergillosis, candidiasis, and mucormycosis (Juvvadi et al., 2019).

## Rapamycin—FKBP12 Interaction in Pathogenic Fungal Species

**FIGURE 4:**
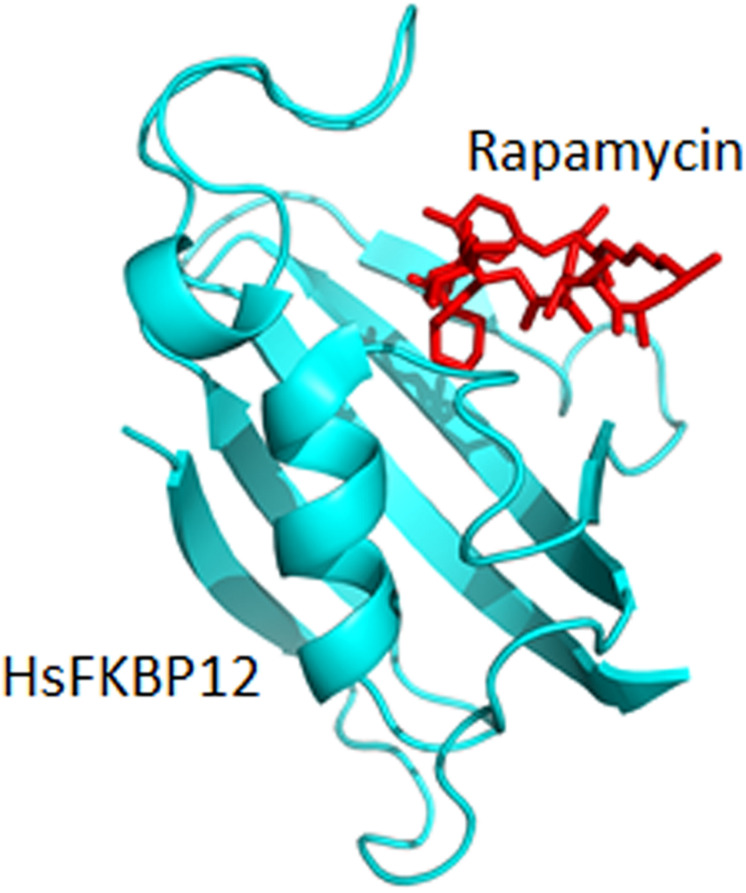
Binding of rapamycin to the bovine FKBP12 (PDB: 1FKL).

The interaction between rapamycin and FKBP12 (Figure 4) is known to result in the inhibition of the TOR pathway in fungi. This inhibition has been known to affect various cellular responses such as protein synthesis inhibition and autophagy (Cardenas et al., 1999; Bastidas et al., 2012). This rapamycin—FKBP12 interaction in pathogenic fungi has become the focus of various research groups as summarized below.

**FIGURE 5:**
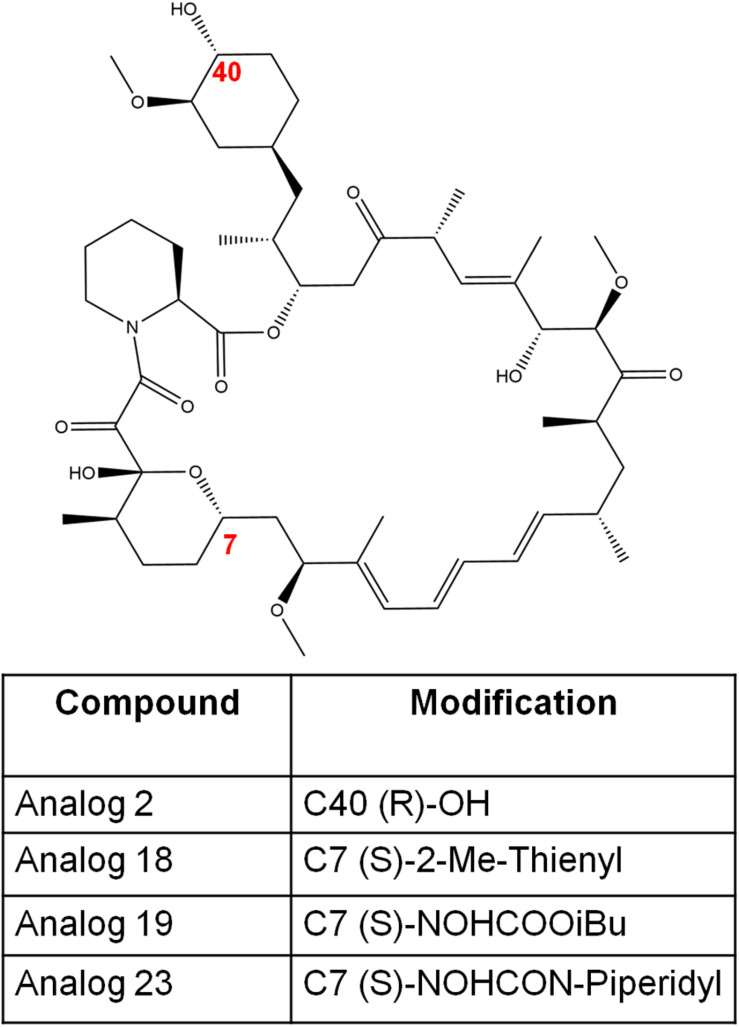
The chemical structures of rapamycin and its analogs in this study. The numbers in red font indicate the modified positions to generate analogs.

### Aspergillus fumigatus

Little is known about the role of TOR in this filamentous fungus, although research has found that there may be a link with mitochondrial processes (Baldin et al., 2015). Specifically, a link between ornithine/arginine biosynthesis and an iron deficiency stress response of *A. fumigatus* have been studied (Baldin et al., 2015). The deletion of the single *tor* gene in *A. fumigatus* results in a lethal phenotype, thus researchers had to develop a conditional lethal *tor* mutant (Baldin et al., 2015). This was achieved by replacing the endogenous *tor* gene by the inducible *xylp-tor* gene cassette (Baldin et al., 2015). Furthermore, the qRT-PCR analysis uncovered an increase in mRNA levels of ornithine biosynthesis genes under iron limitations, and iron regulation was lost when *tor* was repressed (Baldin et al., 2015). Furthermore, the iron regulator HapX was also investigated and researchers found that *hapx* expression was significantly reduced when *tor* was repressed (Baldin et al., 2015). This research concluded that the function of TOR may have two scenarios in *A. fumigatus*; TOR either acts upstream of HapX, or independently of HapX as a repressor of ornithine biosynthesis (Baldin et al., 2015). Finally, the effects of rapamycin on this inducible mutant were investigated. Rapamycin showed strong growth inhibition on the inducible mutant (Baldin et al., 2015). However, the increase of *tor* mRNA levels in the inducible mutant resulted in a slightly increased resistance against rapamycin (Baldin et al., 2015). Previous research also found a single *tor* gene in *Aspergillus nidulans*, a less frequent causal agent of aspergillosis in immunocompromised patients (Loewith and Hall, 2011).

### Candida albicans

The necessity for new or alternative treatments for candidiasis is urgent due to emerging antifungal resistance. Previous studies have deduced that rapamycin presents antifungal activity against *C. albicans* (Sehgal et al., 1975). Rapamycin is known to diffuse into the cell and associate with FKBP12 (Cardenas et al., 1999; Bastidas et al., 2012). The newly formed FKBP12-rapamycin complex will target the two TOR kinases Tor1 and Tor2 (Cardenas et al., 1999; Bastidas et al., 2012). In *C. albicans*, the *RBP1* gene is known to encode a homolog of FKBP12 (Cruz et al., 2001). Interestingly, studies have shown that mutant strains lacking the *RBP1* and *TOR1* genes are viable and rapamycin resistant (Cruz et al., 2001). These findings show that rapamycin antifungal activity is exerted via FKBP12 and Tor1 homologs in *C. albicans* (Cruz et al., 2001). One major contributor to antifungal drug resistance in *C. albicans* is loss of genes encoding ATP-binding cassette (ABC) transporters in the genomes (Lyons and White, 2000; Khandelwal et al., 2018). These transporters help export various substrates across the cell membrane including antifungal drugs (Khandelwal et al., 2018). Previous studies found that the loss of the transporter *CDR6/ROA1* results in a strain that is more resistant to antifungal treatment with azoles (Khandelwal et al., 2018). Researchers developed a *CDR6/ROA1* null strain and found that along with the increase in azole resistance, transcriptional profiling uncovered ribosome biogenesis genes were significantly upregulated (Khandelwal et al., 2018). This finding led to the discovery of a suspected link between ribosome biogenesis and TOR1 signaling (Khandelwal et al., 2018). Furthermore, the mutant strain was grown on media supplemented with rapamycin where TOR1 hyperactivation was observed (Khandelwal et al., 2018). This led to an Hsp90-dependent calcineurin stabilization resulting in an increase to azole resistance (Khandelwal et al., 2018). These findings were then replicated *in vivo* with a systemic infection mouse model, which resulted in a higher fungal load post fluconazole treatment in mice infected with this mutant strain (Khandelwal et al., 2018). This study uncovered a novel mechanism of azole resistance in *C. albicans* involving TOR signaling.

### Cryptococcus neoformans

Although the TOR pathway is a highly conserved mechanism across eukaryotes, its role in *C. neoformans* is a topic of debate (So et al., 2019). Researchers have found two Tor-like kinases, Tor1 and Tlk1, in *C. neoformans* (So et al., 2019). This study discovered that *TLK1* is a dispensable gene resulting in the cultivation of a viable strain following its deletion. The opposite was concluded when *TOR1* was deleted (So et al., 2019). Further research explored the function of Tor1 by creating strains that overexpress the *TOR1* gene (So et al., 2019). It was concluded that Tor1 negatively regulates two crucial virulence factors, DNA damage response, and thermotolerance by reducing Rad53 and Mpk1 phosphorylation, respectively (So et al., 2019). When inhibiting TOR via rapamycin treatment actin depolarization in a Tor-1 dependent manner was observed (So et al., 2019). Finally, various rapamycin-sensitive and resistant strains were screened uncovering that the TOR pathway can possibly crosstalk with various stress signaling pathways (So et al., 2019).

### Mucor circinelloides

One major hurdle encountered by physicians treating mucormycosis patients is that Mucorales are resistant to most antifungal drugs (Caramalho et al., 2017; Dannaoui, 2017). Thus, it has become of great interest to scientists to develop a greater understanding of how this fungus can evade antifungal drugs. One major target of interest is understanding the interaction between rapamycin and FKBP12 in this fungus. A recent study found that the target and mechanism of rapamycin in *M. circinelloides* is mediated via conserved FKBP12 and Tor homologs (Bastidas et al., 2012). In this study researchers utilized spontaneous mutations that disrupted conserved residues in FKBP12, this resulted in rapamycin and FK506 resistance in these mutants (Bastidas et al., 2012). Total disruption of the FKBP12-encoding gene (*fkbpA*) also resulted in rapamycin and FK506 resistance (Bastidas et al., 2012). To further confirm their findings the expression of *M. circinelloides* FKBP12 was complemented in an *S. cerevisiae* mutant strain lacking FKBP12 resulting in the restoration of rapamycin sensitivity (Bastidas et al., 2012). Furthermore, it was found that the *M. circinelloides* FKBP12 and Tor interacted in a rapamycin-dependent fashion (Bastidas et al., 2012). Finally, *in vitro* studies found that rapamycin exhibited potent growth inhibition against *M. circinelloides* followed by an *in vivo* study with *Galleria mellonella* improving survival by 50% post-rapamycin treatment (Bastidas et al., 2012). These findings suggest that rapamycin can be an attractive drug target, and the development of rapamycin analogs with a lowered immunosuppressive activity can have the potential for novel antifungal treatment strategies.

### Novel Small Molecules and Rapamycin Analogs Targeting TOR

We have previously established that the TOR pathway in fungi can regulate various cell stress pathways (Cardenas et al., 1999; Bastidas et al., 2012). This makes the TOR pathway an attractive target for the development of new antifungals. Researchers have previously identified a small molecule—beauvericin that not only potentiated antifungal treatment but was also tolerated by human cells (Shekhar-Guturja et al., 2016). Previous studies have found that beauvericin can enhance azole efficacy against the top leading fungal pathogens *C. albicans, C. neoformans*, and *A. fumigatus* (Shekhar-Guturja et al., 2016). Beauvericin has also shown the ability to block the emergence of resistance and even render resistant strains responsive to treatment (Shekhar-Guturja et al., 2016). The previously mentioned effects have been determined to be mediated via inhibition of multidrug efflux pumps and TORC1 signaling (Shekhar-Guturja et al., 2016). This activates the protein kinase CK2 resulting in the inhibition of the molecular chaperone Hsp90 (Shekhar-Guturja et al., 2016). Furthermore, this research found that substitutions in the efflux transporter Pdr5 that enable beauvericin efflux results in the impairment of azole efflux halting the resistance to this drug combination (Shekhar-Guturja et al., 2016). Similarly, an ATP-competitive TOR kinase inhibitor INK128 has been studied for the possible use of combination therapy with various azoles (Gao et al., 2016). In this study, 23 strains of *Aspergillus* were tested, all of which were clinical isolates derived from patients with invasive aspergillosis (Gao et al., 2016). The function of INK128 involves the inhibitor binding to the TOR catalytic domain and selectively inhibiting TOR (Gao et al., 2016). It was concluded that the efficacy of the azoles tested was significantly increased, although the same was not reported when tested with echinocandins and polyenes (Gao et al., 2016).

The synthesis of rapamycin analogs that present a lowered immunosuppressive activity in humans has been previously explored (Cruz et al., 2001; Guo et al., 2019). One major research involved synthesizing over 45,000 rapamycin-inspired macrocycles (Guo et al., 2019). This massive undertaking was achieved by creating a rapamycin-like macromolecule library by replacing the effector domain of rapamycin with a combinatorial library of oligopeptides (Guo et al., 2019). By the use of ring-closing metathesis, these researchers were able to develop a robust macrocyclization method resulting in the synthesis of a 45,000-compound library of these hybrid macrocycles, named rapafucins, which utilized optomized FKBP-binding domains (Guo et al., 2019). Although this specific study was focused on making rapamycin-like molecules that can bind to new cellular proteins to aid with different types of diseases and disorders; we believe it is important to highlight the potential of synthesizing 45,000 rapamycin-like macrocycles and the possible therapeutic potentials such compounds may possess. Further studies have uncovered a series of rapamycin analogs that present a lowered immunosuppressive activity while presenting antifungal properties in *C. albicans* (Cruz et al., 2001). In this study, four analogs—analog 2, analog 18, analog 19, and analog 23 (Figure 5) were studied to determine their antifungal efficacy compared to rapamycin (Cruz et al., 2001). Previous studies found that the FKBP12 homolog Rbp1 is required for rapamycin antifungal function (Cruz et al., 2001). This was confirmed by developing a *C. albicans rbp1/rbp1* mutant strain lacking a homolog of the FKBP12 protein (Cruz et al., 2001). It was found that these mutants were viable and resistant to both rapamycin and its analogs (Cruz et al., 2001). With regards to the fungicidal activity presented by these analogs, analogs 2 and 23 were found to be toxic and fungicidal to *C. albicans, C. neoformans*, and *S. cerevisiae*, while analogs 18 and 19 were weakly toxic to *C. neoformans* and had no activity against the other fungi tested. In this study, analogs 2 and 23 were found to be the most toxic to *C. albicans* and *C. neoformans* while sharing a common mechanism of action with rapamycin involving an FKBP12-dependent inhibition of the TOR kinases (Cruz et al., 2001).

## Conclusion

Fungal calcineurin and TOR are key targets to develop new antifungals. In this review, we highlighted the structural differences between human and fungal FKBP12, calcineurin, and TOR components. FK506 targets both human and fungal calcineurin. However, several FK506 analogs, particularly which had modifications at C9, C21, C22, and C31 had increased affinity toward fungal FKBP12 and calcineurin resulting in lower immunosuppression while retaining antifungal activity. The sequences of FKBP12, calcineurin, and TOR components vary among fungal species. As a result, the antifungal efficacy of these analogs varies among the fungal pathogens tested. Nevertheless, these studies provide a proof of concept for developing non-immunosuppressive FK506 or rapamycin analogs with antifungal activity.

## Author Contributions

SL and SV conceived the study. SV, AG, and SL wrote the manuscript. All authors contributed to the article and approved the submitted version.

## Conflict of Interest

The authors declare that the research was conducted in the absence of any commercial or financial relationships that could be construed as a potential conflict of interest.
